# Hospital Administration as an Emerging Specialty for Medical Graduates in India: A Cross-Sectional Study

**DOI:** 10.7759/cureus.113848

**Published:** 2026-08-03

**Authors:** Mukunda C Sahoo, Jawahar SK Pillai, Ramkrishna Mondal, Biswajeevan Sahoo, Md Sameer, MV Kiran Kumar, Niranjan Savarirajalu

**Affiliations:** 1 Hospital Administration, All India Institute of Medical Sciences, Bhubaneswar, Bhubaneswar, IND; 2 Hospital Administration, All India Institute of Medical Sciences, Patna, Patna, IND; 3 Hospital Administration, Kurnool Medical College and Government General Hospital, Kurnool, IND; 4 Pharmacology and Therapeutics, Government Medical College, Nagpur, Nagpur, IND

**Keywords:** careers in hospital administration, healthcare administration, hospital administration, hospital administration as a profession, hospital manager, medical administration

## Abstract

Introduction: Most hospitals in India are managed by senior clinicians who are not qualified in hospital administration. The role of qualified hospital administrators does not seem to be highlighted while planning human resources for healthcare services. Postgraduate seats in hospital administration are suboptimal considering the demand. The study aims to understand hospital administration as a specialty for medical graduates and obtain the opinions of healthcare professionals and medical students about hospital administration as a career specialization.

Materials and methods: A cross-sectional study was conducted for 12 months in two phases. Phase 1 comprised qualitative data collection through focused interviews with 25 medical doctors holding administrative responsibilities in hospitals to explore key domains relevant to the study objectives. The findings from these interviews informed the development and refinement of the study. Phase 2 involved a nationwide cross-sectional questionnaire-based survey administered to 574 healthcare professionals.

Results: Phase 1 included focused interviews with medical doctors (n=25) having administrative roles in the hospitals. Subsequently, a pre-tested and pre-validated questionnaire-based national survey (n=574) among the healthcare professionals was conducted in Phase 2. Phase 1 highlighted the role of hospital administration in healthcare services in the Indian scenario, such as hospital operations, planning and designing, human resource management, legal and statutory aspects, quality and patient safety, and others. It also provided an insight into the career prospects and postgraduate courses available for doctors in hospital administration. Phase 2 consisted of a questionnaire-based survey among healthcare professionals, of which 49% were medical students. Using descriptive statistics, it was found that 71% of participants lacked adequate knowledge about hospital administration but were aware of it as a specialty. Only 6% of respondents correctly answered the question concerning hospital administration postgraduate degrees offered in India. Similarly, 56% of participants expressed their willingness to learn more about hospital administration.

Conclusions: The specialty of hospital administration as a postgraduate professional course and the role of a hospital administrator were explored. The study concluded that hospital administration is an essential component for safe and quality healthcare services and necessitates doctors in an administrative role to be qualified in hospital administration. It was evident from the study that hospital administration has highly promising prospects in the future and most participants in the survey considered taking up the specialty as their career subject.

## Introduction

Hospital administration refers to managing and coordinating healthcare facilities, ensuring operational efficiency, and providing quality patient care. Modern hospitals are complex organizations that require qualified hospital administrators with the necessary knowledge and skills to effectively manage the wide range of services and responsibilities involved in healthcare delivery. Healthcare services have undergone a paradigm shift from conventional methods to scientific and evidence-based care supported by technology like clinical decision support systems, artificial intelligence, robotic surgeries, high-end diagnostics, and therapeutics. With technological progress, patients are well-informed about their rights and are concerned about their health. India has witnessed a growing medico-legal burden and increasing awareness of patients' legal rights, resulting in more frequent medical negligence claims under the Consumer Protection Act (CPA) [1]. In most cases, the common reasons for negligence or patient harm are improper documentation, inadequate communication, poorly managed supplies, unsafe practices and facilities, and poor supervision of services [2]. A hospital administrator plays a vital role in preventing such incidents by bringing in standardization of processes, development of manuals or standard operating procedures (SOPs), preventive maintenance, periodic audits, regular staff training, and supervision of support services. This not only improves the healthcare services but also ensures the mitigation of stress for the clinicians.

The specialty of hospital administration in India was started in the 1960s at the All India Institute of Medical Sciences (AIIMS), New Delhi. Marquette University, in Milwaukee, Wisconsin, United States, offered the first-ever hospital management degree program in 1922. Michael Davis [3,4] mentioned a formal course in hospital administration in his book "Hospital Administration: A Career" in the year 1929. Caldwell [5] presented the option of a career in the profession of hospital administration with the objectives of direct and indirect services for patients. Hospital administration emphasizes the practical aspects of running a healthcare institution in partnership with clinicians with teamwork and leadership [6,7]. Healthcare management in India originally developed to meet the administrative needs of the healthcare system [8]. Today, the field faces several critical challenges. These include filling vacant positions, aligning specific jobs with appropriate training, and updating the educational curriculum.

The landmark Bhore Committee report of 1946 in India [9], officially known as the Health Survey and Development Committee, recommended healthcare administration in India. In order to accomplish this, the government took several actions to prepare itself for advancing healthcare management nationwide [10,11]. In 2011, India's High-Level Committee on Universal Health Coverage recommended strengthening management by offering doctors postgraduate courses in public health and hospital administration and creating a specialized state-level management cadre [12]. Historically, India's first official postgraduate degree in this field was the Master in Hospital Administration (MHA), launched at AIIMS, New Delhi, in 1966 [13] and upgraded to an MD in 2016. Many other institutions like the Armed Forces Medical College (AFMC), Pune; Postgraduate Institute of Medical Education and Research (PGIMER), Chandigarh; Nizam's Institute of Medical Sciences (NIMS), Hyderabad; and AIIMS, Bhubaneswar, started the program later.

The National Medical Commission (NMC) reports only 74 postgraduate seats in hospital administration (0.15% of all postgraduate seats) in India to date. Though postgraduate medical specialization is available, several seats in hospital administration have remained vacant in several national institutes in the past years [14]. The practice of engaging senior clinicians in administrative responsibilities in India could be due to insufficient qualified hospital administrators, less awareness about the specialty during undergraduate studies [15], and inadequate professional courses for doctors in hospital administration, thus leading to a gap in the demand and availability of hospital administrators in the country. PGIMER, Chandigarh, is the only institute in India that created the designation Professor of Hospital Administration cum Medical Superintendent [16]. All the AIIMS have faculty positions for hospital administration. Many clinicians take additional courses to learn about this specialty at a later stage in their careers as they realize the importance of hospital administration in managing healthcare facilities. Common causes for patient harm and medico-legal issues can be addressed or prevented by engaging hospital administrators and implementing policy changes [17]. This study highlights the need to expand the specialty and strengthen undergraduate and postgraduate training programs in hospital administration, including the introduction of modules on hospital administration in the Bachelor of Medicine, Bachelor of Surgery (MBBS) curriculum.

The primary objective was to explore the role and career pathway of hospital administration for medical graduates. The secondary objectives of this study were to assess the level of awareness of hospital administration as a medical specialty among healthcare professionals, evaluate their perceptions regarding the scope and roles of hospital administrators, and examine their interest in pursuing hospital administration as a career specialization.

## Materials and methods

This cross-sectional study was conducted over 12 months in two phases at AIIMS, Bhubaneswar, a tertiary care teaching hospital located in Bhubaneswar, India. The Strengthening the Reporting of Observational Studies in Epidemiology (STROBE) format was applied.

Phase 1 consisted of focused interviews with doctors engaged in hospital administration in selected hospitals (September 2024-February 2025). The interviews were based on the research objectives, incorporating open-ended questions to elicit in-depth responses. A purposeful sampling technique was used to select medical doctors with an MD or equivalent degree in hospital administration and holding administration positions as study participants (n=25) working in healthcare institutions that included medical colleges, public hospitals, and corporate or private hospitals. Non-medical administrators were excluded from the study. The interviews were conducted in English language both virtually/online and physically/in-person as applicable, and the observations were scripted by the resident doctors. Prior to the interview, informed consent was obtained, ensuring confidentiality and voluntary participation. Interviews were conducted in a quiet setting and without any leading questions to minimize bias. The interview primarily focused on understanding the specialty of hospital administration and the role of a hospital administrator. It also included participants' qualifications, their study institutions, years of work experience, positions and roles assigned to them, and information on career prospects for a doctor with post-graduation in hospital administration.

Phase 2 (March 2025-August 2025) consisted of a questionnaire-based cross-sectional survey among medical professionals/students of India. A convenience sampling technique was used. The minimum required sample size was calculated using Cochran's formula for an unknown population, yielding a sample size of 385 at a 95% confidence level and a 5% margin of error. To account for an anticipated response rate of approximately 50%, more than twice the required sample (800 medical professionals/students) was invited to participate through student networks, alumni associations, academic institutions, and social media groups. A total of 601 responses were received. After excluding 27 responses due to incomplete questionnaires, duplicate submissions, or invalid entries, 574 complete responses were included in the final analysis, yielding an effective response rate of 71.75%. The study questionnaire was developed using Google Forms (Google LLC, Mountain View, California, United States) based on the study objectives and relevant literature. Content validity was established through review by five subject-matter experts, comprising three faculty members in hospital administration, one medical education expert, and one public health researcher, who evaluated the questionnaire for relevance, clarity, comprehensiveness, and appropriateness of the items. Their feedback was incorporated to refine the questionnaire before pilot testing. The revised questionnaire was pilot-tested among 20 randomly selected medical professionals and medical students to assess clarity, feasibility, and internal consistency. The questionnaire demonstrated acceptable reliability, with a Cronbach's alpha of 0.733. The final questionnaire was administered in English. It focused on awareness of hospital administration as a postgraduate medical degree and as a career specialty. It also included knowledge about available courses in hospital administration, with an open-ended question to understand the willingness of medical graduates to consider hospital administration as their subject specialization. The findings were statistically analyzed using simple statistics in Microsoft Excel (Microsoft Corporation, Redmond, Washington, United States).

Ethical approval

Ethical clearance was obtained for the study from the Institutional Ethics Committee of AIIMS, Bhubaneswar (approval number: T/IM-NF/Hos.Admin/22/138; date: September 2, 2024).

## Results

Phase 1 highlighted the role of hospital administration in healthcare services in the Indian scenario, such as hospital operations, planning and designing, human resource management, legal and statutory aspects, quality and patient safety, and others. It also provided an insight into the career prospects and postgraduate courses available for doctors in hospital administration. Phase 2 consisted of a questionnaire-based survey among healthcare professionals (n=574), of which 49% were medical students. About 71% of participants lacked adequate knowledge about hospital administration but were aware of it as a specialty. Only 6% of respondents correctly answered the question concerning hospital administration postgraduate degrees (NMC recognized) offered in India. Nearly 56% of participants expressed their willingness to learn more about hospital administration. The specialty of hospital administration as a postgraduate professional course and the role of a hospital administrator were explored.

The interview responses from Phase 1 were compiled and manually reviewed by the research team. Similar responses were grouped together to identify recurring patterns and common areas of agreement. These findings were summarized descriptively to develop a list of the key roles and responsibilities of hospital administrators, which subsequently formed the design of the Phase 2 questionnaire. Phase 1 was summarized, and the key roles of hospital administration were identified in Table 1.

**Table 1:** Roles of the hospital administrators

Key aspects	Brief description of the key roles
Hospital operations	Supervise all activities that are necessary to ensure the efficient delivery of all the healthcare services in the hospital. This includes outpatient departments, inpatient departments like wards and ICUs, specialized units like operation theatres, dialysis, blood transfusion, labor and maternity services, emergency services, etc. along with support services like diagnostic services (laboratory and radiology), pharmacy services, stores and procurement, engineering services like civil, electrical, and air-conditioning, medical gases, sanitation, waste management, etc.
Human resources	This deals with planning of manpower requirements like faculty members, resident doctors, nursing personnel, and allied healthcare professionals, recruitment process, promotion, performance appraisal, adequate staffing, and training and development of the staff
Strategic planning and designing of hospitals	Hospital administration is involved in long-term planning to set organizational goals, define the mission and vision, and develop strategies to achieve them such as the expansion of services, upgradation of units, development of new facilities, etc. based on the demand for healthcare services
Financial management	Planning of the hospital's budget, costing of services, controlling costs, pricing, and billing processes, overseeing revenue cycles, sources of funds, and financial sustainability
Facility management	Includes support services like housekeeping and engineering services like civil, electrical, and air-conditioning to provide a safe and comfortable environment for patients and staff
Legal and regulatory compliance	Hospitals need to adhere to various laws, regulations, and standards. Administrators need to stay informed about healthcare policies, licensing requirements, and accreditation standards to ensure compliance
Patient safety and quality assurance	Achieving accreditation is essential to preserving and raising the standard of healthcare services. This involves implementing quality improvement initiatives, monitoring patient outcomes, and addressing issues to enhance overall patient care
Information technology	Hospital administration also involves the implementation and maintenance of healthcare information systems, electronic health records, artificial intelligence, telemedicine, and other technology solutions to streamline operations and enhance patient care
Community relations	Building and maintaining positive relationships with the community, patients, and other stakeholders is an essential part of hospital administration. This includes communication, outreach programs, and community engagement initiatives
Risk management	Identifying and managing potential risks to patient safety, data security, and other areas is a crucial responsibility. Administrators work to develop and implement risk management strategies
Emergency preparedness	Hospitals must be prepared for emergencies and disasters. Hospital administrators are involved in developing and implementing emergency response plans to ensure the safety of patients and staff
Ethics	Hospital administrators ensure that the organization operates ethically with social responsibility

**Figure 1:**
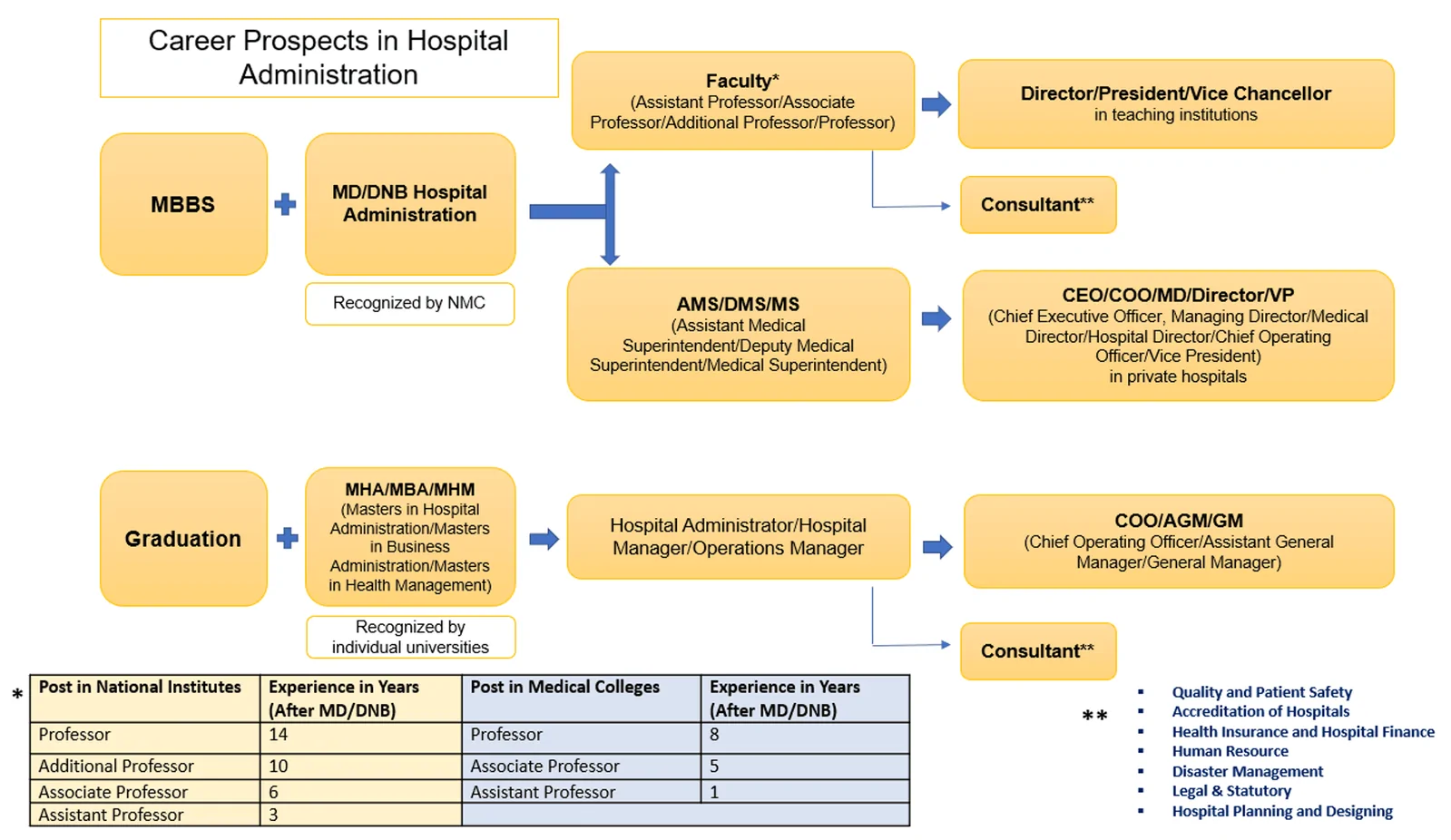
Career aspect in hospital administration MBBS: Bachelor of Medicine, Bachelor of Surgery; MD: Doctor of Medicine; DNB: Diplomate of National Board; NMC: National Medical Commission Different career path aspects are described after graduation and post-graduation in the field of hospital administration. This figure was created using Microsoft PowerPoint (Microsoft Corporation, Redmond, Washington, United States).

The career prospects for candidates are varied as per the type of organization (Figure 1).

**Figure 2:**
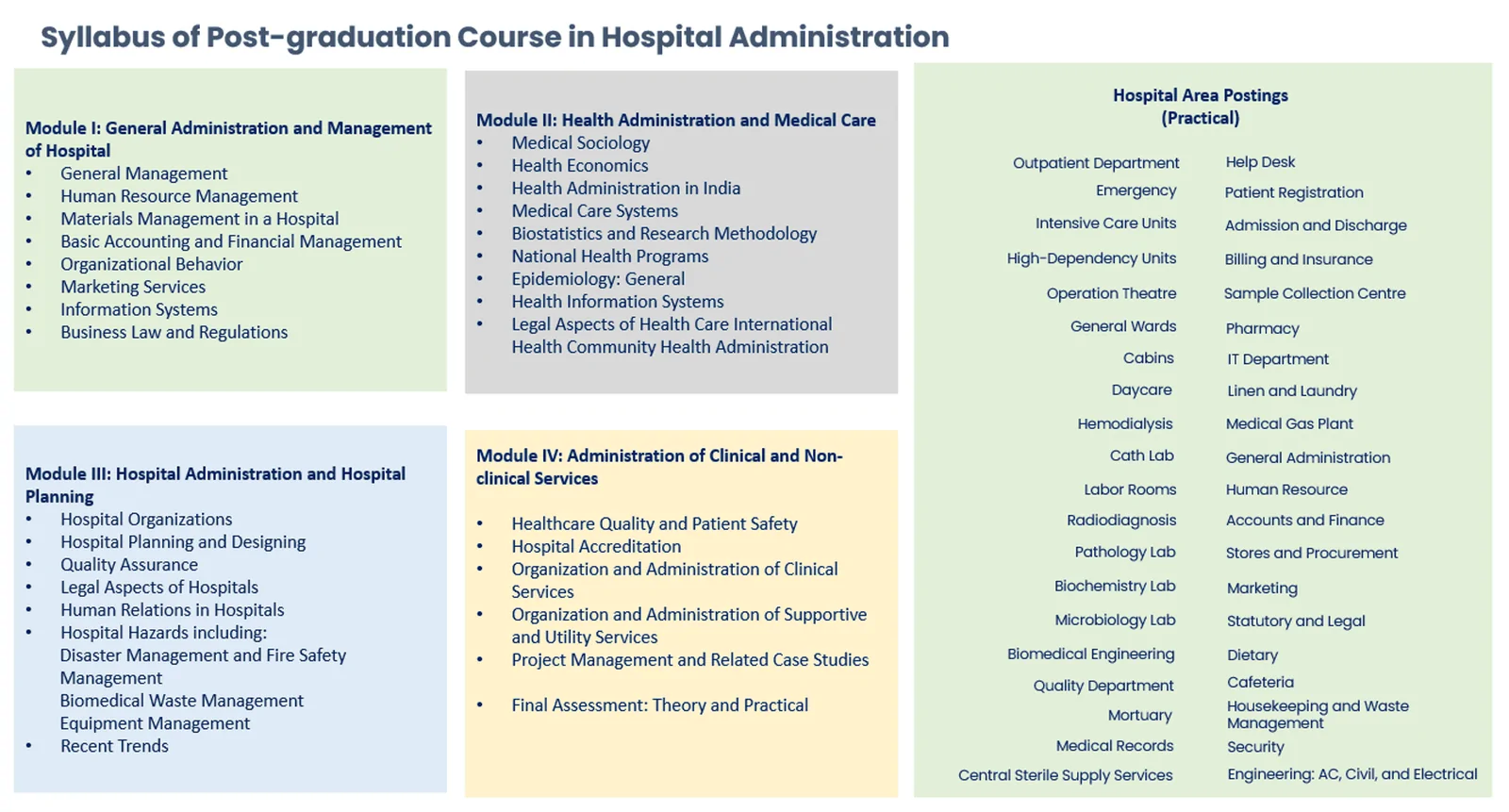
Brief curriculum of postgraduate course in hospital administration The curriculum of post-graduation course syllabus in hospital administration is briefly described. This figure was created using Microsoft PowerPoint (Microsoft Corporation, Redmond, Washington, United States).

In teaching medical institutions, qualified professionals can be faculty members, medical superintendents (MS), deans, directors, or representatives in various advisories and government bodies, whereas in public sector hospitals, they can be posted as assistant MS, deputy MS, medical superintendent, or chief medical officer. In the case of private sector hospitals, the career starts with operational managers or medical administrators and can be promoted to medical superintendents, medical directors, and chief executive officers. In addition, one can opt to become an independent consultant or an expert in various domains of hospital administration. The postgraduate course is offered in 20 medical institutions, including AIIMS in India. Any medical graduate can qualify in this specialty through national-level entrance exams. The duration of the course is three years. Figure 2 represents a sample MD hospital administration course curriculum.

The MD program is special because it puts students in direct contact with hospitals and gives them hands-on experience managing hospital services by integrating them into routine chores like the control room. The MD program provides hands-on practical training in decision-making, delegation, crisis management, situational leadership, out-of-the-box thinking, and emergency preparedness. The National Board of Examination (NBE) offers postgraduate specialization (DNB) in hospital administration, and the candidates can register themselves with NMC. Several other programs of two-year and one-year duration are offered by some institutions in full-time/part-time mode for any medical and non-medical graduates. Such graduate courses are recognized by the individual universities and not by the NMC and are also less preferred at the senior level by the hospitals as compared to MD graduates. Doctors as hospital administrators are different from the managers and are most accepted as leaders with authority in the medical community.

**Figure 3:**
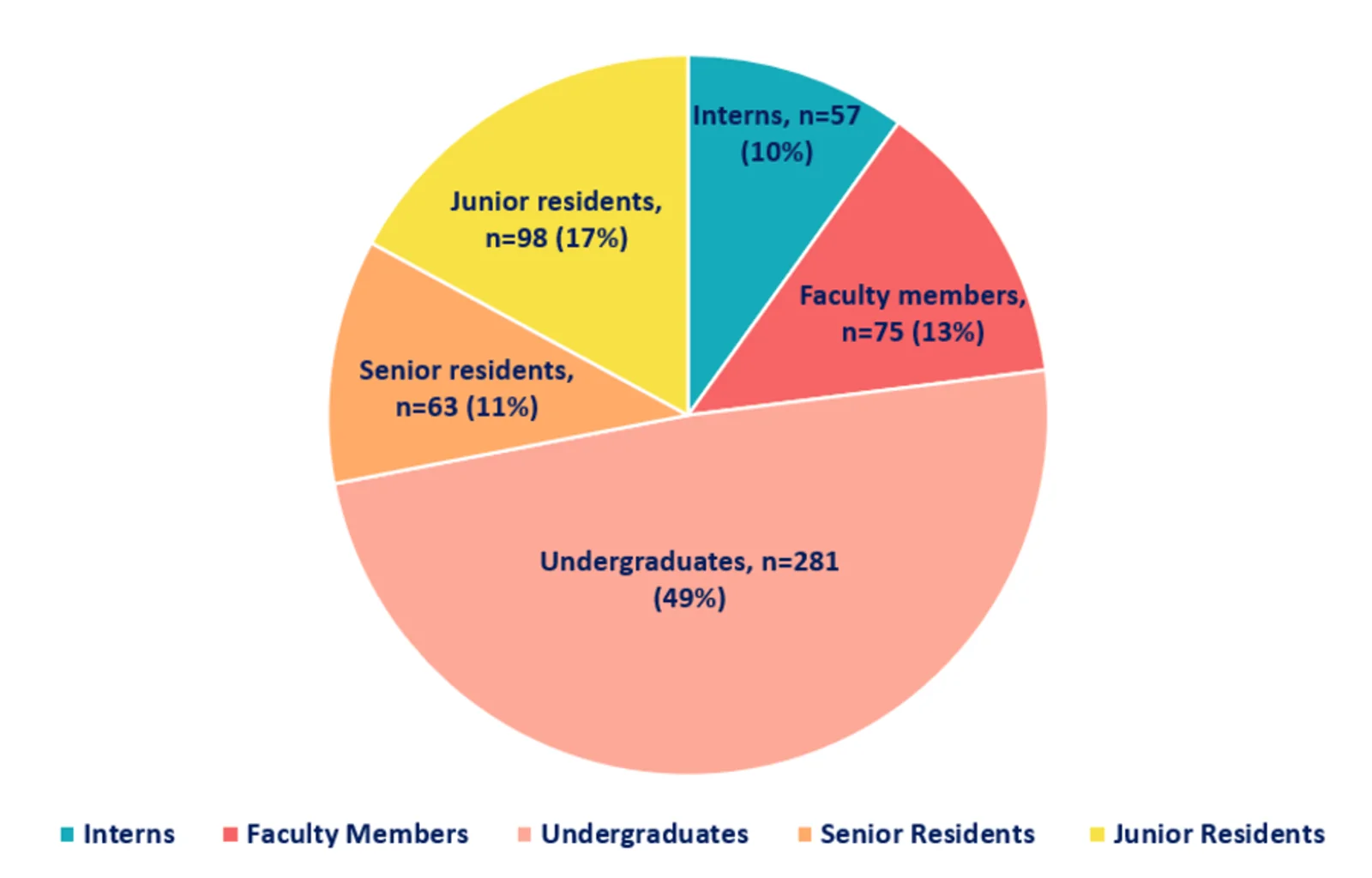
Different categories of survey participants Different categories of participants who participated in the survey: undergraduates, n=281 (49%); interns, n=57 (10%); junior residents, n=98 (17%); senior residents, n=63 (11%); and faculty members, n=75 (13%). This figure was created using Microsoft PowerPoint (Microsoft Corporation, Redmond, Washington, United States).

In Phase 2, 574 responses were received in the survey as shown in Figure 3.

Male participants comprised 49% of the population, females comprised 49%, and 2% of people withheld their gender. Around 50% of the population was between the ages of 18 and 20, followed by those between the ages of 21 and 25 (29%). Table 2 summarizes the responses to the survey.

**Table 2:** Responses of the study participants Question-wise responses of the study participants except question number 2 as it is a dependable variable. MCI: Medical Council of India; NMC: National Medical Commission

Question no.	Question	Positive or correct or yes (%)	Negative or incorrect or no (%)	I do not know or not sure (%)
1	Are you aware of hospital administration as a specialty or department?	71	23	6
3	Are health administration and hospital administration the same?	13	61	26
4	Is there any difference between hospital management and hospital administration?	65	8	27
5	Is hospital administration a medical specialty?	44	32	24
6	What are the degrees available in India for hospital administration?	6	52	42
7	Who can study hospital administration as a specialty?	38	28	34
8	Is the hospital administration course an MCI-/NMC-recognized degree?	50	5	45
9	When did the hospital administration course start in India?	24	24	52
10	In your opinion, what are the prospects of hospital administration?	56	22	22
11	If you get a chance to study hospital administration, are you willing to study?	56	19	25

About 71% of participants were aware of hospital administration as a specialization. Around 35% of respondents mentioned that their sources of knowledge were friends and co-workers, whereas only 1% came from media sources. While 61% of respondents believed that there was a distinction between hospital and health administration, 13% disagreed, and 26% were unsure. Only 6% of respondents correctly answered the question concerning hospital administration degrees offered in India. Although 50% of respondents believed that NMC recognizes hospital administration as a specialty, 5% responded incorrectly, and 45% were unsure. Nearly 56% of participants expressed their willingness to learn about hospital administration.

The responses to the open-ended question were grouped into four categories (Table 3).

**Table 3:** Some relevant comments from the respondents Some of the specific and relevant responses in the form of comments obtained from different participants.

Category (n; %)	Responses
Relevant (13; 12%)	I am interested in managing hospitals as a healthcare service provider.
	I think it will be helpful if I want to operate my hospital.
	It is a respected area of interest, and a good administrator can transform healthcare in their area of influence.
	The course is structured and disciplined and has a promising future in the Armed Forces with this degree.
	There is a need for mentoring and guidance about the specialty to select it as a career pathway.
Not relevant (12; 11%)	Equal opportunity should be given to all specialties to strengthen hospital services.
	A person with basic technical knowledge of healthcare infrastructure and services can study hospital administration.
	No proper cadre of posts is available in the country, and there is no individual decision-making in hospital administration.
Less awareness or no information (52; 46%)	Unclear about prospects after studying hospital administration.
	Do not know if it plays well or gets the same respect as the clinical doctor.
	Depends on the facilities, awareness of the course, and personal interest.
	Any qualified doctor can do it, in my opinion.
	Experience is required before joining the MD course; details of this subject should be included in the MBBS curriculum.
	Never heard of hospital administration as a specialty or career path for the medical practitioner.
	Not oriented, never taught or informed about hospital administration in any class or workshop.
Not interested (36; 32%)	After finishing my MBBS, I have a passion for treating patients.
	It is my dream to practice medicine.
	Wish to pursue other clinical specialties rather than hospital administration.

The first category comprised 12% of the relevant responses considering hospital administration as a career option. The second category grouped the responses that were not relevant to the specialty. The third category had 46% of respondents who had less or no knowledge about the specialty. The fourth category had 32% of respondents who were aware of the specialty but were not interested in pursuing hospital administration as a career. There was a positive correlation between the 18-20-year age group and choosing the specialty as a career option if they get an opportunity (r=0.51 and p<0.05 at 95% CI).

## Discussion

Despite the visionary recommendations of the Bhore Committee, the specialty of hospital administration has not been developed in India as expected, so as to manage all the hospitals. The number of medical institutions in India with a Department of Hospital Administration is 0.03% of the total 706 medical colleges [18].

The interviews with experts provided a comprehensive insight into the role of a hospital administrator, which includes hospital operations, human resources, strategic planning and designing of hospitals, financial management, facility management, legal and regulatory compliance, patient safety and quality assurance, information technology, community relations, risk management, emergency preparedness, ethics, etc., and career prospects are also exceptionally good. The majority of the study participants (79%) were below 25 years, with approximately half of them being medical graduates. Though two-thirds of the participants were aware of the specialty of hospital administration, half of them did not know about the postgraduate programs in hospital administration and the associated career prospects. However, more than half of the participants agreed that the prospects of hospital administration are good.

The study highlighted the possible growth opportunities for medical graduates who select this specialization after completing their primary medical degree. The course contents seem to be more managerial instead of clinical, thereby inferring that being a hospital administrator requires managerial and leadership skills, healthcare knowledge, and business acumen. Hospital administrators are trained to navigate a complex and dynamic healthcare environment to provide optimal patient care while managing the various challenges associated with healthcare delivery. Considering the paradigm shift in healthcare, such as rising hospital expenditure, an increased awareness of patients and expectations, health insurance, an increase in litigation, violence against doctors and hospitals, etc., efficient management of hospitals is the need of the hour. Therefore, qualified and experienced hospital administrators should be employed in all hospitals so that there are professionally qualified and trained individuals who can dedicate themselves full-time to the smooth functioning of the hospital. However, such a scenario is rare in the country and is limited to institutes like AIIMS, New Delhi, and PGIMER, Chandigarh.

On the other hand, the availability of trained manpower is also suboptimal. The present practice of engaging senior clinicians to take responsibility for hospital administration needs to be re-examined. Thus, there is a need to strengthen the Department of Hospital Administration in all medical colleges and institutes in the country and to develop postgraduate programs in hospital administration for training the budding medical graduates. States like Kerala, Andhra Pradesh, Telangana, Jammu, and Kashmir have taken steps in the development of the specialty in the health sector, like starting the department in all medical colleges, instituting an administrative cadre, etc.

Regarding the implementation of quality and safety improvement programs in hospitals, a hospital administrator is the primary driver for patient care initiatives by developing SOPs, manuals, and clinical pathways, standardizing processes, performing clinical audits, and evaluating patient feedback and grievances. They can coordinate and integrate hospital services to match the intangible needs of patients, which are often not visualized by the clinical care team. The guidelines and standards developed by the National Accreditation Board for Hospitals and Healthcare Providers (NABH) have brought in standardization of healthcare services, and hospital administrators are key elements towards the successful implementation of the same in hospitals [19].

Healthcare providers have realized the impact of quality and safety programs on patient outcomes and are undertaking accreditation programs by national and international bodies. Antimicrobial resistance, infectious disease-related mortalities, and morbidities are typical burning issues in any nation's healthcare sector. These issues demand management of hospitals by hospital administrators who can manage these issues with a multidisciplinary approach. India's hospital market was valued at US$ 98.98 billion in 2023 and is projected to grow at a compound annual growth rate (CAGR) of 8% from 2024 to 2032, reaching an estimated value of US$ 193.59 billion by 2032 [20]. Therefore, the medical community needs to pay greater attention to this specialization to adequately handle the rapid expansion of the healthcare sector in India and other nations. Hospital administrators can significantly contribute to accomplishing the goal of having a strong healthcare workforce. It is imperative to note that the healthcare industry is growing fast and is among the top professions globally [21-23].

Sharma and Zodpey [24] studied the demand and supply in human resource capacity for hospital management in India. They opined that there is a lack of trained hospital managers and administrators to work for hospitals. They identified that about 2500 hospital management professionals are qualified annually, whereas there is an estimated requirement of more than 21000 professionals based on the country's present healthcare growth status. Sachdeva and Sachdev [25] mentioned the skills and practices of the postgraduate trainees in hospital administration courses in India that enable them to perform their present duties effectively and prepare them to shoulder greater responsibilities in the future. Similar to the present study, they identified the list of essential management skills for the students in hospital administration that included planning, organizing, staffing, training, decision-making, controlling, supervision, budgeting, proposals, medical audits, statistical data management, research, etc. Tiwari et al. [26] studied the supply and need gap for health management professionals in India by analyzing the various academic programs related to health administration and ongoing public health programs. They mentioned that the availability of trained health administrators in 2017 was 3463 compared to the required number of 11304. They also projected the need for about 45000 management professionals by 2030. Devnani [27] mentioned that clinical doctors hold most leadership positions in hospitals in India due to an inadequate health management workforce. Clinicians lack formal education and training in management, policy, and leadership. He opined that medical professionals opting for formal education in management sciences and taking up leadership positions in health systems are expected to bring a sea change in the healthcare delivery service.

The World Health Organization speaks about the lack of leadership and management capacity as a constraint on the efficient operation of public and private healthcare sectors despite the resources spent by the governments to strengthen these capacities [28]. Lal, way back in 1998, mentioned the need for health management courses in the undergraduate medical curriculum in his editorial. The need for preparing doctors to assume leadership roles is widely felt in the current scenario, and the willingness of doctors to assume such roles needs to be investigated [29]. The emphasis should be on generating awareness about the benefits of hospital administration specialties with clear career objectives. Collaborative efforts from healthcare leaders and policymakers to prioritize the development of capable hospital administrators will ensure better patient outcomes that remain at the forefront of healthcare administration, ultimately shaping the future of healthcare for generations to come [30].

Future work with strengths and limitations

The study design is unique as it approached the doctors involved in hospital management to explore the role of hospital administration and also collected the opinions of medical professionals at a national level about careers in hospital administration. There have been very few comparable studies carried out in India. The study can be expanded to a larger number of medical students and institutions. The prospects of a career in hospital administration at an international level could not be evaluated in this study and are considered a limitation. The exclusion of non-medical professionals engaged in hospital management is also a limitation in addition to convenience sampling, possible response bias, limited representativeness, etc.

Policy recommendations

The authors recommended the following strategies expected to improve healthcare services in India in the future. First, modules of hospital administration are to be included in the undergraduate medical (MBBS) curriculum in India with topics like an overview of support services, quality, safety, hospital infection control, accreditation, antimicrobial stewardship program, etc. Second, a Department of Hospital Administration may be started in all government and private medical colleges, and the NMC should consider including it in all medical colleges in India. Third, the department is to be strengthened in all medical institutions with adequate faculty members, and operations of hospital services should be entrusted to the specialty. Fourth, qualifications of MD-HA/MHA are to be made essential for recruitment to the post of medical superintendent in all medical institutions.

## Conclusions

Hospital administration is an essential component for safe and quality healthcare services and necessitates doctors in an administrative role to be qualified in hospital administration. It was evident from the study that hospital administration has highly promising prospects in the future and most participants in the survey considered taking up the specialty as their career subject. The specialty of hospital administration as a postgraduate course and the role of hospital administrators were explored. The study concluded that qualified professionals in hospital administration are vital for delivering safe and high-quality healthcare services. Appropriate mentorship during the undergraduate medical course can generate awareness of the specialization among medical students and thus reduce the associated apprehensions about the prospects of the subject specialty.
